# Utilization of spontaneous breathing trial, objective cough test, and diaphragmatic ultrasound results to predict extubation success: COBRE-US trial

**DOI:** 10.1186/s13054-023-04708-y

**Published:** 2023-10-31

**Authors:** Fabio Varón-Vega, Luis F. Giraldo-Cadavid, Ana María Uribe, Adriana Rincón, Jonathan Palacios, Stephanie Crevoisier, Eduardo Tuta-Quintero, Lina Ordoñez, Natalia Boada, Paola Rincón, Marcela Poveda, Pablo Monedero

**Affiliations:** 1 Critical Care and Lung Transplantation Service, Fundación Neumológica Colombiana, Fundación Cardio Infantil, Bogotá, Colombia; 2 Critical Care Service, Fundación Neumológica Colombiana, Fundación Cardio Infantil, Cra. 13B #161 - 85, 110131 Bogotá, Colombia; 3 Interventional Pulmonology Service, Fundación Neumológica Colombiana, Bogotá, Colombia; 4 School of Medicine, Universidad de La Sabana, Chía, Colombia; 5 Critical Care Service, Fundación Clínica Shaio, Bogotá, Colombia; 6 School of Medicine, Universidad de Navarra, Pamplona, Spain

**Keywords:** Airway extubation, Cough, Mechanical ventilation, Diaphragm

## Abstract

**Background:**

The results of clinical and weaning readiness tests and the spontaneous breathing trial (SBT) are used to predict the success of the weaning process and extubation.

**Methods:**

We evaluated the capacity of the cuff leak test, rate of rapid and shallow breathing, cough intensity, and diaphragmatic contraction velocity (DCV) to predict the success of the SBT and extubation in a prospective, multicenter observational study with consecutive adult patients admitted to four intensive care units. We used receiver operating characteristic (ROC) curves to assess the tests’ predictive capacity and built predictive models using logistic regression.

**Results:**

We recruited 367 subjects who were receiving invasive mechanical ventilation and on whom 456 SBTs were performed, with a success rate of 76.5%. To predict the success of the SBT, we derived the following equation: (0.56 × Cough) − (0.13 × DCV) + 0.25. When the cutoff point was ≥ 0.83, the sensitivity was 91.5%, the specificity was 22.1%, and the overall accuracy was 76.2%. The area under the ROC curve (AUC-ROC) was 0.63. To predict extubation success, we derived the following equation: (5.7 × SBT) + (0.75 × Cough) − (0.25 × DCV) − 4.5. When the cutoff point was ≥ 1.25, the sensitivity was 96.8%, the specificity was 78.4%, and the overall accuracy was 91.5%. The AUC-ROC of this model was 0.91.

**Conclusion:**

Objective measurement of cough and diaphragmatic contraction velocity could be used to predict SBT success. The equation for predicting successful extubation, which includes SBT, cough, and diaphragmatic contraction velocity values, showed excellent discriminative capacity.

**Supplementary Information:**

The online version contains supplementary material available at 10.1186/s13054-023-04708-y.

## Background

Invasive mechanical ventilation (IMV) is used to facilitate gas exchange and reduce or replace the physical effort required to breathe in critically ill patients in intensive care units (ICUs) through the use of an external mechanical system [1, 2]. The criteria for applying mechanical ventilation are widely described in the medical literature and are set by clinical management guidelines and consensus [3]. When a patient’s clinical condition stabilizes, the weaning process (WP) is initiated. However, it poses risks for different populations, such as older people who are hospitalized in an ICU for a prolonged period and people with chronic respiratory or neuromuscular diseases. This underscores the importance of determining the probability of success or failure of the WP before making the crucial decision to initiate the process [4–6].

The WP involves gradually decreasing ventilatory support, the continual stabilization of respiratory failure, the recovery of oxygenation indices (e.g., the ratio of the partial pressure of arterial oxygen to the fraction of inspired oxygen (PaO2/ FiO2)) a reduced need for FiO2, improvement in other laboratory test results, and an objective clinical assessment [7, 8]. The WP can be classified as simple, difficult, or prolonged. A successful WP results in extubation and no ventilatory support for up to 48 h after extubation, whereas the WP is considered a failure when the patient fails the spontaneous breathing trial (SBT), requires reintubation within 48 h of extubation, or dies within 48 h of extubation [7, 9]. Early weaning preparation and a systematic approach to IMV interruption can increase the incidence of successful weaning to 30–60% in high-complexity centers [7, 8]. Failure of this process can lead to reintubation, which carries a high risk of morbidity and mortality and increased healthcare costs [1, 2, 7].

Successful extubation (SE) is defined as the absence of a need for IMV for more than 48 h after extubation and depends on several factors, including the general condition of the patient, the control of the underlying disorder, the ventilation–perfusion ratio, the cough and expectoration capacity, lung compliance, and diaphragmatic function [7, 10, 11]. Therefore, it is crucial to assess these factors in order to determine the optimal timing for extubation and thus avoid extubating too early or unnecessarily prolonging the IMV, as well as complications and unfavorable clinical outcomes [6, 11, 12]. Recently, several tests have emerged as possible predictors of extubation outcome; however, none have shown outstanding performance [1, 2, 6, 8]. In real-world scenarios, practitioners have been found to employ certain techniques in an attempt to predict the probability of SE, such as the SBT using a T-piece or pressure support ventilation with low levels of ventilatory support [4, 13]. However, there is limited evidence of the success of this approach, other variables and tests associated with extubation failure have not been considered, and the published clinical results are unsatisfactory due to the multifactorial nature of extubation failure [4, 5, 8, 13].

Ultrasound has also been proposed as a valuable tool for assessing diaphragm morphology and function in this context [14–16]. The diaphragm is the most important muscle in the respiratory cycle. Diaphragmatic dysfunction that is characterized by a loss of strength related to the use of a ventilator [15] occurs in up to 20% of patients who experience difficult weaning and leads to a potential 50% increase in the duration of mechanical ventilation [14–16]. Two characteristics measured during diaphragmatic ultrasound have been identified as predictors of extubation failure: diaphragmatic excursion, with a sensitivity of 75% and a specificity of 75% to predict failure; and the fraction of diaphragm thickening, with a receiver operating characteristic curve (ROC curve) value of 0.87 to predict failure or success [16, 17].

Despite significant technological advancements and extensive clinical research, certain limitations persist that make it challenging to improve the success rates of weaning and extubation. These limitations impact crucial factors such as survival rates, hospitalization duration, and financial burdens [6, 8, 14, 15]. Hence, we developed and tested the following hypothesis in this study: combining the results of weaning readiness tests that are readily available within each ICU will enhance the ability to predict weaning outcomes. Therefore, our objective was to evaluate the predictive capacity of the following weaning readiness tests for SBT and extubation success in patients undergoing IMV: cuff leak test, rapid and shallow breathing index (RSBI), cough intensity, and diaphragmatic ultrasound. Additionally, we developed and internally validated a multivariate predictive model using the variables found to be associated with SBT and extubation success. This model can be used to aid decision-making about critically ill patients in ICUs.

## Methods

### Study period, location, and participants

We conducted a prospective, multicenter observational study from February 2019 to November 2021. The study sample consisted of consecutive adult patients admitted to four ICUs located at Fundación Cardioinfantil, Fundación Neumológica Colombiana, and Fundación Clínica Shaio in Bogotá, Colombia. The Institutional Review Boards of the participating institutions approved this study, and informed consent was obtained from the patients’ relatives.

### Definition of the main objectives

We divided the WP into two parts: the SBT and SE. Our primary aim was to identify predictive variables for SE, defined as the absence of death or the need for reintubation within 48 h of extubation [7–9]. Our secondary aim was to develop a predictive model for SBT success (see criteria below).

### Eligibility criteria

**Fig. 1:**
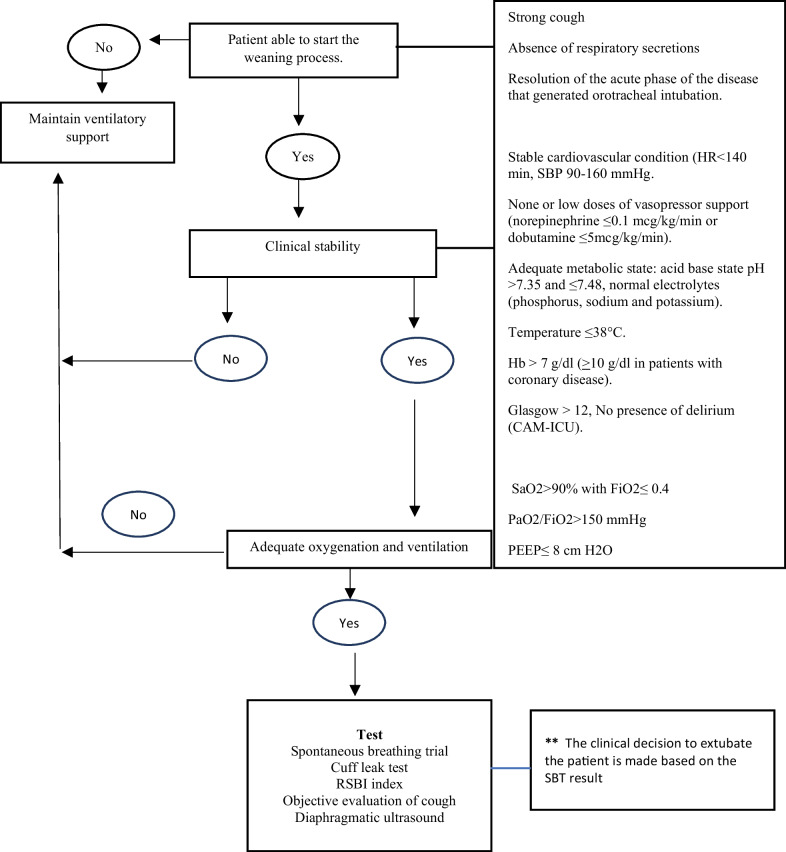
Criteria for initiating the weaning process. *SBP* systolic blood pressure, *HR* heart rate, Hb, hemoglobin, *ICU* intensive care unit, *CAM-ICU* confusion Assessment method for the ICU, *SaO2* oxygen saturation, *FiO2* Fraction of Inspired Oxygen, *PaO2/FiO2* ratio of partial pressure of oxygen in arterial blood to the fraction of inspiratory oxygen concentration, *PEEP* Positive End-Expiratory Pressure, *RSBI* rapid shallow breathing index

Eligible participants included adults who required IMV for more than 48 h and met the criteria for starting the WP, which included general condition, clinical stability, and adequate oxygenation, as described in Fig. 1. Patients with acute brain injuries, neurosurgical patients, pregnant women, and patients with neuropsychiatric diseases or diaphragmatic paralysis were excluded from the study.

### Clinical tests

Each patient underwent a 30-min SBT using a T-piece or pressure support ventilation. The trial was suspended in cases of intolerance to the test. Failure was defined as the presence of at least one of the following criteria: partial pressure of arterial oxygen (PaO2) ≤ 60 mmHg or the arterial blood oxygen saturation (SpO2) ≤ 90% with the fraction of inspired oxygen (FiO2) ≥ 0.50, PaCO2 > 50 mmHg or an increase of > 8 mmHg from baseline, pH < 7.32 or a decrease of > 0.7 units, respiratory rate ≥ 35/min or an increase of ≥ 50% from baseline, heart rate ≥ 140 bpm or an increase of ≥ 20% from baseline, systolic arterial pressure > 180 mmHg or an increase of ≥ 20% or systolic arterial pressure < 90 mmHg. Additional criteria included the development of de novo cardiac arrhythmias, an abrupt change in mental status, and the presence of two or more signs of respiratory distress, such as tachycardia, bradycardia, increased breathing effort, use of accessory muscles, abdominal paradox, facial signs of distress, diaphoresis, cyanosis, and marked dyspnea [7, 13, 18].

The cuff leak test was performed to determine the difference in expiratory tidal volume with the cuff inflated and deflated. During testing, the patient was on the ventilator set to support control mode at a tidal volume of 10–12 mL/kg. The patients were not administered any sedative medications during the test, only analgesic medications. One inspiratory tidal volume and six subsequent expiratory tidal volume values were recorded after oropharyngeal suctioning and endotracheal tube cuff deflation. Cuff leak was measured as the difference between the preset inspiratory tidal volume and the average of the lowest three of six subsequent expiratory tidal volume values. A larger leak value has been shown to predict SE, and a favorable test result was defined as a difference greater than 120 mL [19, 20].

The RSBI index was calculated as the ratio of tidal volume to respiratory frequency (f/VT) and assessed immediately after the cuff leak test during the transition to spontaneous mode. A value lower than 105 breaths/min/L was considered a favorable test result [17, 21].

To objectively evaluate cough, the ventilatory parameters were set to spontaneous mode with zero assistance. Normal 0.9% saline (2 mL) was instilled through the closed suction at the end of inspiration, and the maximum expiratory flow was measured during the resulting involuntary cough. Cough-induced peak expiratory flow was categorized as follows: 0 = no cough, 1 = audible movement of air through the orotracheal tube but no audible cough, 2 = strong cough with mobilization of secretions within the orotracheal tube, and 3 = strong cough with mobilization of secretions out of the orotracheal tube [22].

A diaphragmatic ultrasound was performed to measure the diaphragmatic excursion, duration of the diaphragmatic cycle, diaphragmatic thickening index, and diaphragmatic contraction rate [23–25]. The measurements were performed in the supine position using a subcostal or intercostal approach in the midclavicular or anterior axillary lines. The laterality chosen to perform the measurement was dependent on the ultrasound assessor’s technical ease and clinical decision. Eight ultrasound assessors (medical doctors who had completed a residency or fellowship in critical care medicine and had relevant training and experience) conducted the ultrasound examinations in the ICUs. To acquire competency in diaphragmatic ultrasound, each assessor received two months of chest ultrasound training during a radiology rotation, followed by 8 h of theoretical and practical diaphragmatic ultrasound training for the study measurements. The assessors were then evaluated by an intensive care medicine physician with extensive experience in diaphragmatic ultrasound (FVV) to confirm their competency in the measurement of diaphragmatic variables. This team of assessors previously participated in a similar study two years before this study [16]. After their initial training, the assessors acquired additional expertise in the two years prior to the current study by performing approximately 25 diaphragmatic ultrasounds for ventilator weaning assessments each month in each ICU, totaling approximately 2,400 diaphragmatic ultrasounds per ICU in the two-year period. Each of the assessors performed at least 50 diaphragmatic ultrasounds before participating in the current study. Furthermore, they underwent regular retraining in diaphragmatic variable measurements every six months throughout the study, surpassing previous studies’ requirements and learning curves [15, 23, 26–29].

Diaphragmatic ultrasound values were obtained from three consecutive tidal breaths, and the average values were used in the analysis. Diaphragmatic thickness was measured during quiet spontaneous breathing and during maximal inspiratory and expiratory efforts. An index of diaphragmatic thickening, the thickening fraction (TF), was calculated using the M mode and the following equation: TF = (thickness at end-inspiration − thickness at end-expiration)/thickness at end-expiration. In the M mode, the diaphragmatic excursion (displacement, cm), diaphragmatic contraction velocity (DCV) (slope, cm/s), inspiratory time (Tinsp, s), and duration of the cycle (Ttot, s) were measured as described elsewhere [25, 30, 31].

### Clinical variables

Information was collected on other variables, including age, sex, admission diagnosis, etiology of respiratory failure, arterial gases prior to extubation (e.g., pH, PCO2, HCO3, PO2, FiO2), ventilatory mode during the WP (pressure support or T-piece), duration of the WP (time from the beginning of the WP or change of ventilatory mode to final extubation), days from admission to the ICU until the onset of the WP, and total number of days in the ICU. Patients at risk of WP failure and extubation failure were managed with non-invasive mechanical ventilation or the administration of high-flow nasal cannula oxygen. All patients received clinical care that was in accordance with local medical practice. Data were collected using Research Electronic Data Capture (REDCap) software [32] and included physiological and ventilatory WP variables obtained from medical records.

### Statistical analysis

Continuous variables are presented as means and standard deviations (SDs) or medians and interquartile ranges (IQRs), depending on their distribution, while categorical variables are presented as absolute and relative frequencies. The distribution of continuous variables was assessed using the Shapiro–Wilk test. The t-test or Mann–Whitney U test was used for normally distributed or non-normally distributed variables, respectively. Categorical variables were compared using the χ^2^ test or Fisher’s exact test, as appropriate, based on the frequencies in the contingency table. The reliability of the new tests, such as the objective measurement of cough, was assessed using the intraclass correlation coefficient. As the reproducibility of diaphragmatic ultrasound has already been assessed in a similar setting and population to ours [15, 23, 26–29] and our ultrasound assessors had valid training, we did not test the diaphragmatic ultrasound reproducibility in this study. A two-tailed *P* value less than 0.05 was considered statistically significant.

We assessed the capability of quantitative variables to predict SBT and extubation success by constructing ROC curves. Variables were deemed to possess discriminative capacity when their area under the ROC curve (AUC-ROC) was statistically significant (*p* < 0.05) and exceeded 0.5, indicating their ability to distinguish between successful and unsuccessful outcomes. We used univariate binary logistic regression analysis to assess the association between the variables with discriminative capacity and successful SBT or extubation outcomes. Subsequently, we constructed saturated multivariate logistic regression models to predict these outcomes, and these included all variables with discriminative capacity and significant associations. We constructed two multivariate logistic regression models: SBT success as a dichotomous dependent variable (Eq. 1) and extubation success as a dependent dichotomous variable (Eq. 2). Non-significant independent variables that did not substantially influence the models’ explanatory power (R2) were eliminated from the saturated models for parsimony. We assessed the models’ discriminative capacity using the C statistic via ROC curve analysis and verified their calibration using the Hosmer–Lemeshow test [33]. For each patient, we calculated a score on the scale by multiplying regression coefficients by corresponding variables and summing these values. We derived diagnostic accuracy statistics, including values for sensitivity, specificity, likelihood ratios (LRs), and selected optimal cutoff points using the Youden Index.

Furthermore, an internal validation of the scale was performed using the bootstrap method with replacement, and the AUC-ROC values were recalculated to verify their similarity to those obtained with the original database. The interaction was evaluated by creating new variables equal to the multiplication of the independent variables with potential interaction. The number of interactions to explore was determined using the following equation: ID × ID − 1)/2, where ID corresponds to the number of independent variables in the model. For example, in a model with 2 IDs, 1 interaction was explored: 2 × (2 − 1)/2 = 1; whereas in a model with 3 IDs, 2 interactions were explored: 2 × (3 − 1)/2 = 2.

A sample size of 400 SBTs was estimated for a final model with eight variables and an estimated prevalence of weaning failure of 20%. We used multiple imputation for missing values, with a maximum of 9.5% missing values. All analyses were conducted using Stata version 16 (StataCorp LLC, College Station, USA).

## Results

### Patient characteristics and test scores

A total of 367 patients who received IMV in an ICU were included in this study. Table 1 presents the main characteristics of the enrolled patients. During the study period, 456 SBTs were performed, with a success rate of 76.5%.

**Table 1:** Characteristics of the patients

Number of patients *n* (%)	367 (100)
Male, *n* (%)	219 (59.7)
Age in years, median (IQR)	61 (49–72)
Weight Kg, median (IQR)	70 (60–80)
Height cm, mean (SD)	163.6 (10)
Body mass index kg/m^2^, Median (IQR)	25.3 (21.7–29.1)
Active Smoking, *n* (%)	33 (9)
Active alcoholism, *n* (%)	22 (6)
Reason for ICU admission, *n* (%)	
Medical	345 (94)
Surgical (only postsurgical)	22 (6)
Type of ventilatory failure, *n* (%)	
Shock	52 (14.9)
Hypercapnia (pH < 7.25, high CO_2_)	23 (6.6)
Hypoxemia (PaO_2_ < 60, usual FiO_2_)	261 (75)
Neuromuscular	2 (0.6)
Perioperative	10 (2.9)
Immunosuppression, *n* (%)	72 (19.6)
Pharmacological, *n* (%)	25 (6.8)
HIV, *n* (%)	10 (2.7)
Others, *n* (%)	63 (17.2)
Comorbidities, *n* (%)	
Diabetes Mellitus	113 (30.8)
Hypertension	173 (47.1)
Asthma	8 (2.2)
Pulmonary fibrosis	6 (1.6)
Chronic kidney disease	69 (18.8)
Chronic liver disease	17 (4.6)
Neoplasms, *n* (%)	
Hematological	7 (1.9)
Solid neoplasms	2 (0.5)
Solid neoplasms Chest	1 (0.3)
Solid neoplasms Abdomen	9 (2.4)
Solid musculoskeletal neoplasms	4 (1.1)

*IQR* interquartile range, *SD* standard deviation, *Kg* kilograms, *cm* centimeters, *ICU* intensive care unit. *CNS* central nervous system

The objective measurement of cough, cuff leak test, RSBI, and diaphragmatic ultrasound results are described in Table 2. Only 26 patients had an RSBI > 105. Among the 349 patients in whom the SBT was a success and who were extubated, 31 (8.9%) required reintubation within 48 h. The reproducibility of the objective measurement of cough results, as assessed using the intra-rater intraclass correlation coefficient, was 0.94 (95% confidence interval [95%CI]: 0.89–0.96; *p* < 0.001), indicating almost perfect agreement. The inter-rater intraclass correlation coefficient was 0.72 (95% CI 0.51–0.85; *p* < 0.001), indicating substantial or good concordance. The univariate analyses conducted with the potential predictor variables are described in the Additional file 1: Univariate analysis for predictors of a successful spontaneous breathing trial.

**Table 2:** Weaning readiness tests results

Successful spontaneous breathing trial, *n* (%)	349/456 (76.5)
Objective measurement of cough, *n* (%)	
0	6/451 (1.3)
1	34/451 (7.6)
2	151/451 (33.5)
3	260/451 (57.6)
Cuff leak test, ml, median (IQR)	238 (193–327)
RSBI, median (IQR)	56 (43–75)
Displacement diaphragmatic excursion cm, median (IQR)	1.9 (1.4–2.5)
Diaphragmatic inspiratory time s, median (IQR)	0.8 (0.62–1.01)
Duration of the diaphragmatic cycle s, median (IQR)	2.3 (1.8–2.9)
Diaphragmatic thickening fraction %, Median (IQR)	37 (20–52)
Velocity of diaphragmatic contraction cm/s, median (IQR)	2.3 (1.6–3.8)

*IQR* interquartile range, *RSBI* rapid shallow breathing index, *cm* centimeters, *s* seconds

0 = No presence of cough

1 = Audible movement of air through the orotracheal tube, but no audible cough

2 = Strong cough with mobilization of secretions inside the orotracheal tube

3 = Strong cough with mobilization of secretions outside (expels) the orotracheal tube

**Fig. 2:**
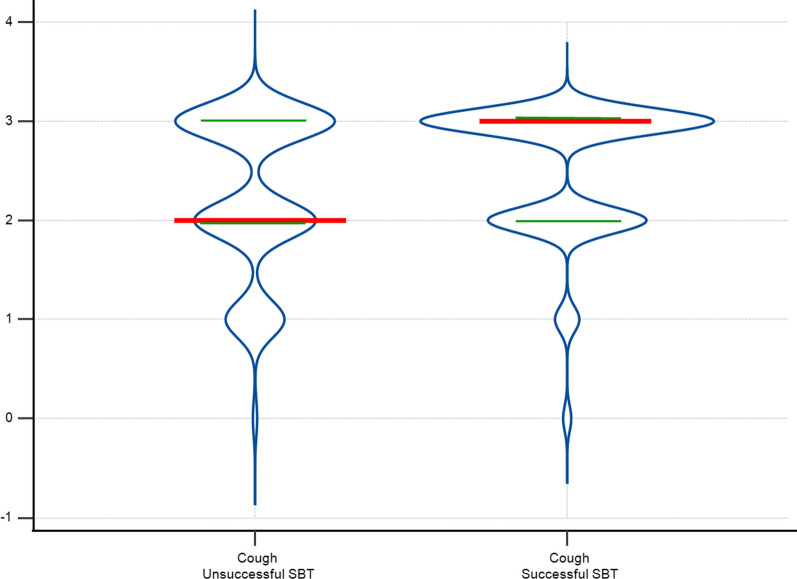
Violin plot of the objective cough measurement by spontaneous breathing test (SBT) success. Violin plot illustrating objective cough measurement data distribution within successful and unsuccessful Spontaneous Breathing Test (SBT) groups. The density curves visually represent data point frequency across the distribution range. In this figure, the width of each curve is proportionate to the approximate frequency of data points within the corresponding region. Red lines represent median values for each group, while green lines denote the 25th and 75th percentiles

We found a significant association between SBT success and the objective measurement of cough (*p* = 0.02) (Fig. 2), DCV (*p* = 0.01), and the duration of the diaphragmatic cycle (*p* = 0.008). Among these three variables, only the objective measurement of cough (odds ratio [OR] 1.68; 95% CI 1.48–1.90; *p* < 0.001) and DCV (OR 0.88; 95% CI 0.83–0.94; *p* < 0.001) were significantly associated with SBT success in the multivariate logistic regression model. The model yielded the following equation:

1 $$(0.56 \times {\text{cough}}) - (0.13 \times {\text{DCV}}) + 0.25.$$

**Fig. 3:**
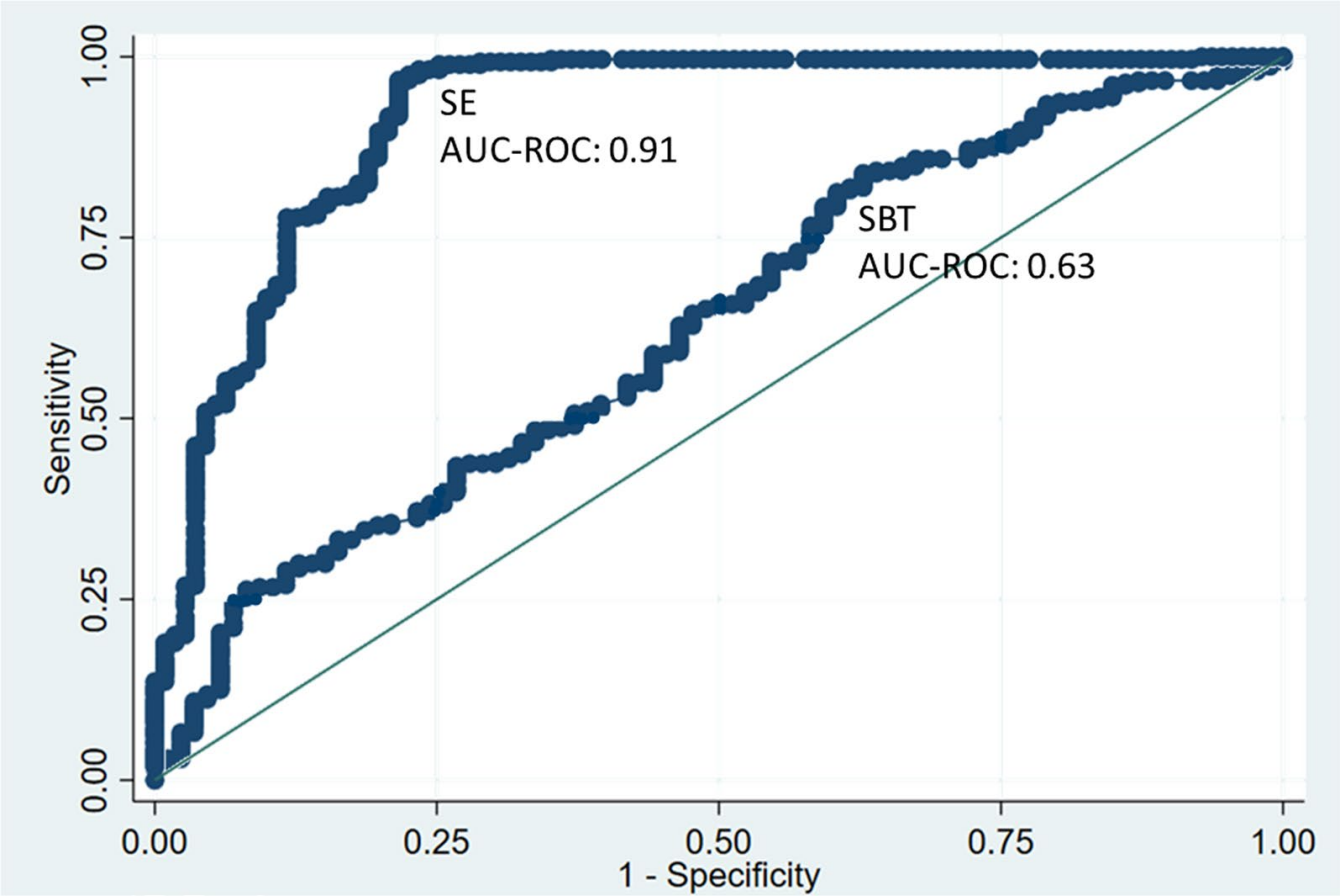
ROC curve of the successful extubation and successful breathing trial prediction model. *SE* successful extubation, *SBT* spontaneous breathing trial success, *AUC-ROC* area under the ROC curve

In this model, cough was rated as a variable that ranged in value from 0 to 3 (see methods), and DCV was a continuous variable (see methods). The AUC-ROC value of this model to predict successful SBT was 0.63 (95% CI 0.56–0.69; *p* < 0.001) (Fig. 3). According to the Youden Index, the cutoff point that resulted in the highest discriminative capacity was ≥ 1.03, yielding a sensitivity of 83.9% and a specificity of 37.2%. However, in the clinical practice of intensive care medicine, a model with higher sensitivity—that can better detect those patients with a greater chance of SBT success—may be more useful. Therefore, we adjusted the cutoff point to ≥ 0.83, and the resultant sensitivity was 91.5%, specificity was 22.1%, LR + was 1.2, LR– was 0.4, diagnostic odds ratio (DOR) was 3.0, and overall accuracy was 76.2%. The Hosmer–Lemeshow test was conducted, and the results showed that there was no significant difference between the observed and predicted values (*p* = 0.17), indicating a good calibration of the model. We found no significant interaction or collinearity between the variables of the predictive model for SBT success (see Additional file 1: Interaction and collinearity analysis).

**Fig. 4:**
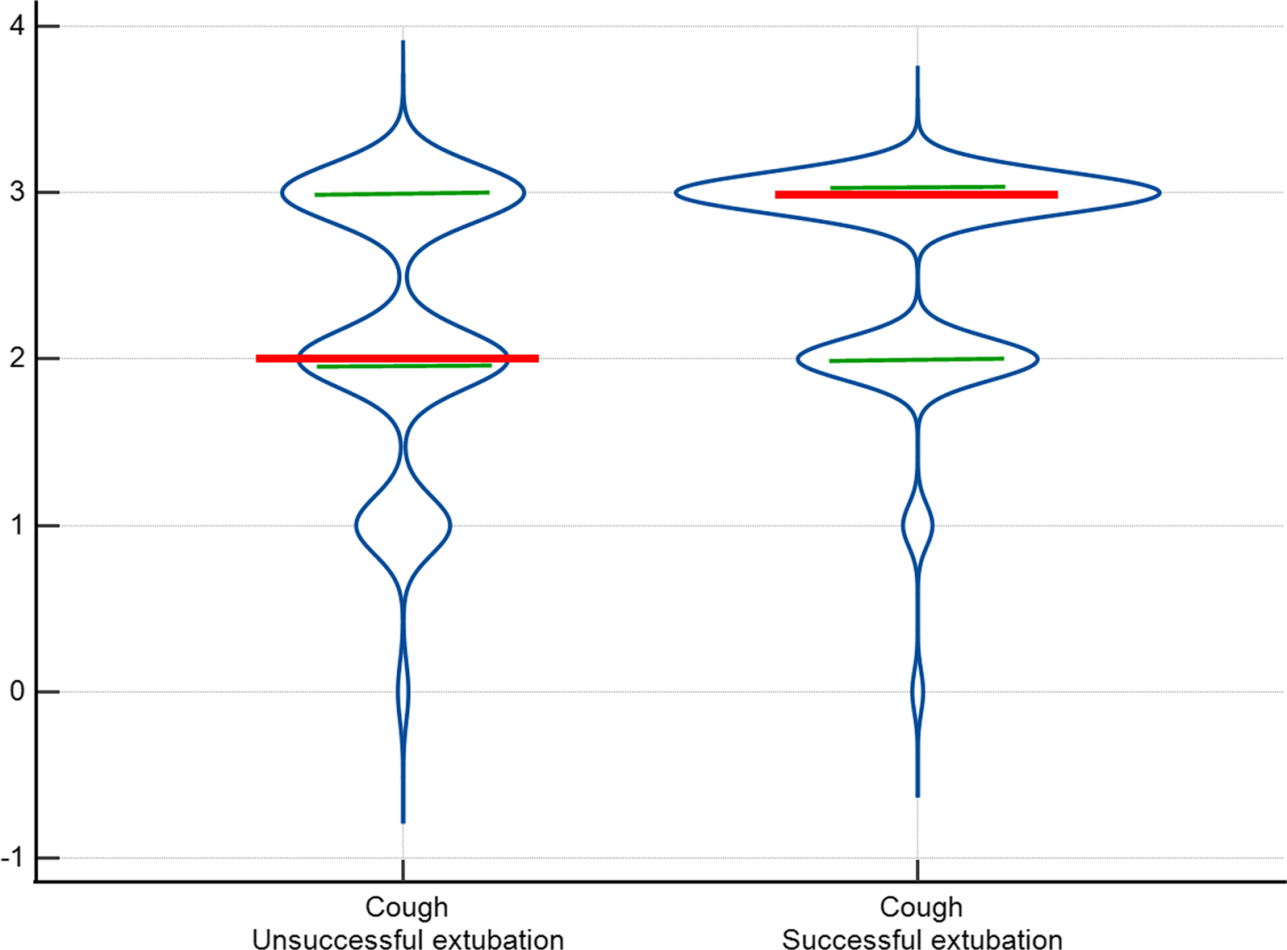
Violin plot of the objective cough measurement by extubation success. Violin plot illustrating objective cough measurement data distribution within successful and unsuccessful extubation groups. The density curves visually represent data point frequency across the distribution range. In this figure, the width of each curve is proportionate to the approximate frequency of data points within the corresponding region. Red lines represent median values for each group, while green lines denote the 25th and 75th percentiles

We also found a significant association between SE and SBT as dichotomous variables (OR 167.0; 95% CI 64–436; *p* < 0.001), and between SE and objective measurement of cough (OR 1.90; 95% CI 1.43–2.54; *p* < 0.001) (Fig. 4), DCV (OR 0.85; 95% CI 0.73–0.99; *p* = 0.04), cuff leak test (OR 1.002; 95% CI 1.0004–1.004; *p* = 0.02), and inspiratory total time (OR 2.1; 95% CI 1.14–3.87; *p* = 0.02) as continuous variables. Among these variables, only SBT, objective measurement of cough, and DCV were significantly associated with SE in the multivariate logistic regression model. The model yielded the following equation:

2 $$(5.7 \times {\text{SBT}}) + (0.75 \times {\text{Cough}}){-}(0.25 \times {\text{DCV}}){-}4.5.$$

In this equation, SBT was rated as a dichotomous variable (1 = successful SBT, 0 = unsuccessful SBT), cough was rated as a variable that ranged in value from 0 to 3 (see methods), and DCV was rated as a continuous variable (see methods). The AUC-ROC value of this model to predict SE was 0.91 (95% CI 0.87–0.95; *p* < 0.001), indicating an excellent discriminatory capacity (Fig. 3). According to the Youden Index, the cutoff point with the highest discriminative capacity was ≥ 1.25. Using this cutoff yielded a sensitivity of 96.8%, a specificity of 78.4%, an LR + of 4.5, an LR − of 0.04, a DOR of 108.8, and an overall accuracy of 91.5% for predicting SE. The Hosmer–Lemeshow test results showed that there was no significant difference between the observed and predicted values (*p* = 0.92), indicating a good calibration of the model. We found no significant interaction or collinearity between these variables of the predictive model for SE (see Additional file 1: Interaction and collinearity analysis). 

## Discussion

The findings of this study contribute to our understanding of factors associated with SE and the ability to predict SBT success. The findings indicate that there is a significant association between objective measurements of cough and DCV and the success of an SBT. Furthermore, objective measurements of cough and DCV were also found to be significantly associated with SE, as was SBT success. Thus, the integration of our predictive models into clinical practice could enhance clinicians’ ability to identify patients who are likely to undergo successful SBTs and extubation, which would ultimately improve the success rate of the WP. However, it is crucial to emphasize that further refinement and validation of our models are essential to optimize their clinical utility.

Most of the studies conducted in this area have focused on determining a patient’s suitability for an SBT individually; to our knowledge, no proposed model combines the results of the multiple tests included in our models [9–11, 14, 15]. Here, we have demonstrated that when cough, which is often subjectively assessed by clinicians, is objectively measured during the WP and the results are combined with diaphragmatic ultrasound results (as described in Eq. 1), the outcome can be used to discriminate—to some extent—patients who are likely to have a successful SBT. The predictive model for a successful SBT, derived from multivariate logistic regression analysis, exhibited limited discriminatory capacity, suggesting that it can poorly predict the success of an SBT. Consequently, it is apparent that the SBT cannot be fully replaced by alternative objective measures based on these results.

During WP the use of clinical tests, severity scores, hemodynamic conditions, and ventilator weaning tests that require patient input to improve the discriminatory ability of the SBT may be limited by factors such as residual sedative effects, delirium, or patient understanding [22, 23, 30]. In terms of extubation success, we have identified several significant predictors: SBT, objective measurement of cough, and DCV. The multivariate logistic regression model for predicting SE that incorporates these variables showed excellent discriminatory capacity. The good calibration of the model, as indicated by the non-significant Hosmer–Lemeshow test result, further supports its validity [33].

In this study, both T-piece and pressure support ventilation were utilized during the SBTs, as they have been shown to have comparable predictive power for SE in critically ill patients [34]. Extubation fails in approximately 20% of extubated patients. Our findings revealed that while the SBT is the best predictor of SE, the objective measurement of cough and DCV also significantly correlated with extubation success. Although the results of the cough test are useful for facilitating extubation decisions, it is rarely used in a standardized manner [35]. Here, we have successfully standardized the objective measurement of cough, ensuring its validity and reproducibility among observers.

Diaphragmatic ultrasound has been examined in multiple studies to determine its usefulness in predicting extubation success, yielding conflicting results [14–16]. However, a common factor among such studies is that the DCV appears to be the measure most strongly correlated with SE [23, 24]. In our study, cough and DCV showed associations with extubation success, although they exhibited less discriminative capacity than the SBT. However, when we included the SBT, DCV, and objective measurement of cough in our model (Eq. 2), the discriminative capacity significantly improved for predicting extubation success.

### Limitations

This was not a clinical study, and the findings may have been influenced by confounding bias. Although the sample was representative, the data must be evaluated in the context of a prospective, multicenter observational study. Also, while internal validation was performed, external validation has not yet been conducted.

The strengths of this study lie in its prospective and multicenter design, its rigorous methodology, the frequency of the diaphragmatic ultrasounds and the associated calculation of average values, the validation and reproducibility of the objective measurement of cough, and the use of tests that can be performed without the patient’s collaboration. This study was further strengthened by the fact that all the tests were performed by trained intensive care personnel and that the transthoracic ultrasounds were performed at the bedside by experienced physicians.

However, since we focused on the ultrasound test that was supported by the most evidence of validation at the time of data collection [16, 23–25, 30, 31], a cutaneous marker was not applied to minimize the variability of the ultrasound measurements, as described by Goligher et al. [23]. In addition, advanced tools that are used to evaluate diaphragmatic function, such as tissue Doppler and speckle tracking, were not utilized [36–39].

## Conclusions

Our findings indicate that when cough and diaphragmatic contraction velocity are objectively measured, the values can be used to predict whether an SBT will be successful or not. In addition, we have formulated an equation using SBT, cough test, and diaphragmatic contraction velocity values that shows excellent discriminative capacity in terms of predicting SE. Hence, our proposed model could be used to determine the probability of achieving SE. However, further research is needed to refine and validate our models in clinical trials with larger study population sizes.

### Supplementary Information

**Additional file 1**. Interaction and collinearity analysis.

## Data Availability

The datasets generated during and/or analyzed during the current study are available from the corresponding author on reasonable request.
