# Supplementary material for: Utilization of spontaneous breathing trial, objective cough test, and diaphragmatic ultrasound results to predict extubation success: COBRE-US trial
Source: Crit Care. 2023 Oct 31;27:414. doi: 10.1186/s13054-023-04708-y (PMC10617213; doi:10.1186/s13054-023-04708-y)
Supplement: Supplementary file 1 — Additional file 1. Interaction and collinearity analysis. [file 13054_2023_4708_MOESM1_ESM.pdf]

Supplementary appendix. Univariate analysis for predictors of a successful spontaneous breathing trial.

Objective measurement of cough

OBS	Area	Std. Err	95% conf. Interval
**451**	0.58	0.03	0.52 - 0.64

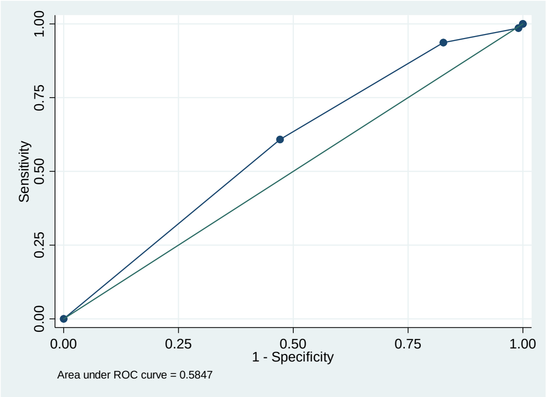

p = 0.002

OR 1.74 95%CI 1.12 – 2.70; P = 0.015

Velocity of diaphragmatic contraction (VDC)

OBS	Area	Std. Err	95% conf. interval
**391**	0.55	0.03	0.49 0.62

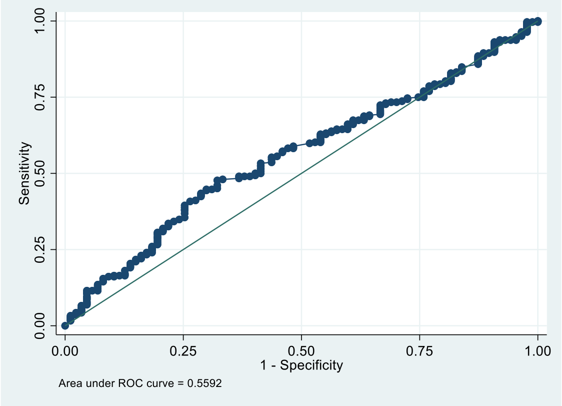

p = 0.04

OR 0.52 95%CI 0.31 – 0.86; P = 0.011

Duration of the diaphragmatic cycle

OBS	Area	Std. Err	95% conf. Interval
**386**	0.57	0.03	0.50 0.64

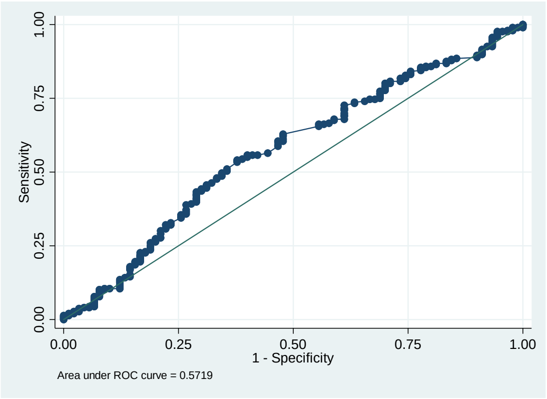

p = 0.019

OR 1.94 95%CI 1.19 – 3.14; P = 0.008

Leak test:

OBS	Area	Std. Err	95% conf. Interval
**444**	0.51	0.01	0.49 - 0.54

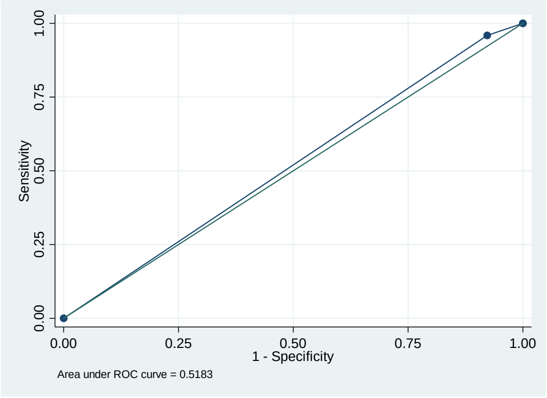

p = 0.100

The AUC-ROC was 0.51 with a confidence interval spanning the null value (0.5) and a non-significant difference from 0.5 (p = 0.100). This indicates that the leak test lacks discriminative capacity to predict success in the spontaneous breathing trial.

Rapid and shallow breathing index (RSBI):

OBS	Area	Std. Err	95% conf. Interval
**447**	0.53	0.03	0.46 - 0.59

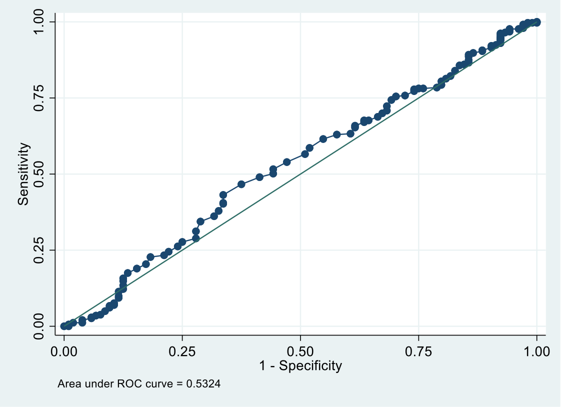

p = 0.161

The AUC-ROC was 0.53 with a confidence interval spanning the null value (0.5) and a non-significant difference from 0.5 (p = 0.161). This indicates that the RSBI lacks discriminative capacity to predict success in the spontaneous breathing trial.

Inspiratory total time:

OBS	Area	Std. Err	95% conf. Interval
**410**	0.55	0.03	0.48 0.62

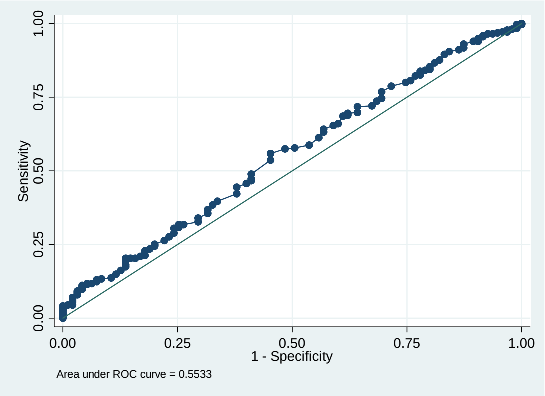

p = 0.057

The AUC-ROC was 0.55 with a confidence interval spanning the null value (0.5) and a non-significant difference from 0.5 (p = 0.057). This indicates that the inspiratory total time lacks discriminative capacity to predict success in the spontaneous breathing trial.

Diaphragmatic excursion

OBS	Area	Std. Err	95% conf. Interval
**418**	0.48	0.03	0.41 - 0.54

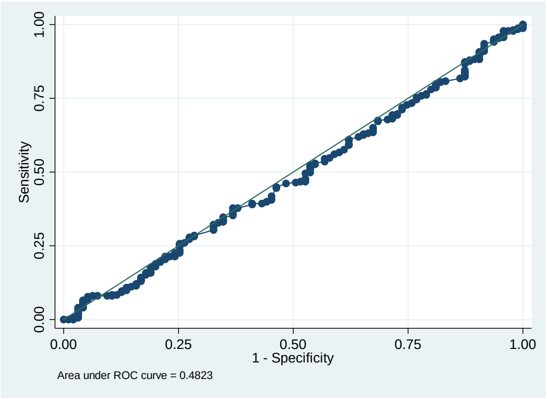

p = 0.300

The AUC-ROC was 0.48 with a confidence interval spanning the null value (0.5) and a non-significant difference from 0.5 (p = 0.300). This indicates that the diaphragmatic excursion lacks discriminative capacity to predict success in the spontaneous breathing trial.

Diaphragmatic thickening index

OBS	Area	Std. Err	95% conf. interval
**353**	0.51	0.03	0.43 0.58

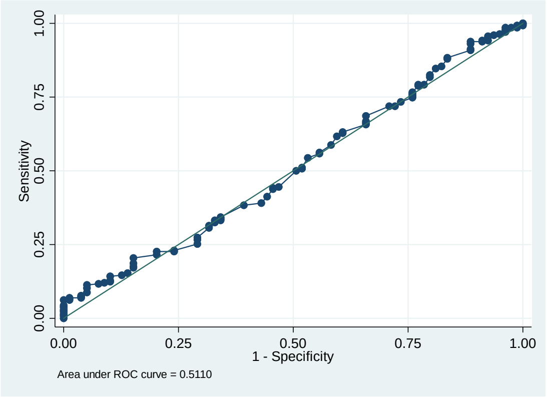

p = 0.384

The AUC-ROC was 0.51 with a confidence interval spanning the null value (0.5) and a non-significant difference from 0.5 (p = 0.384). This indicates that the diaphragmatic thickening index lacks discriminative capacity to predict success in the spontaneous breathing trial.

Supplementary file 1b. univariate analysis for predictors of a successful extubation

Spontaneous breathing trial (SBT)

OBS	Area	Std. Err	95% conf. Interval
**456**	0.86	0.019	0.82 0.89

p < 0.001

OR 167.0 95%CI 64.0 – 436.0; P < 0.001

Objective measurement of cough

OBS	Area	Std. Err	95% conf. Interval
**451**	0.61	0.02	0.56 0.66

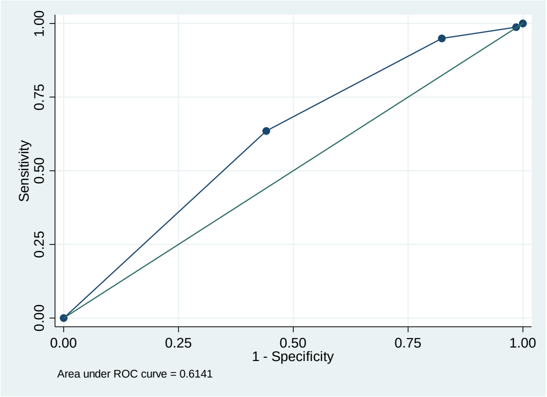

p < 0.001

OR 1.90 95%CI 1.43 – 2.54; P < 0.001

Velocity of diaphragmatic contraction (VDC)

OBS	Area	Std. Err	95% conf. interval
**391**	0.58	0.03	0.52 - 0.64

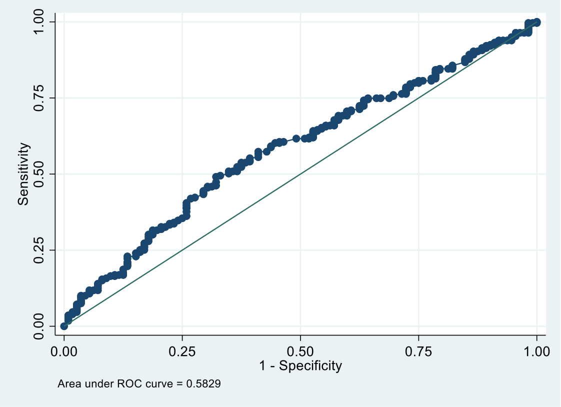

p = 0.004

OR 0.85 95%CI 0.73 – 0.99; P = 0.04

Leak test:

OBS	Area	Std. Err	95% conf. Interval
**441**	0.56	0.03	0.50 - 0.62

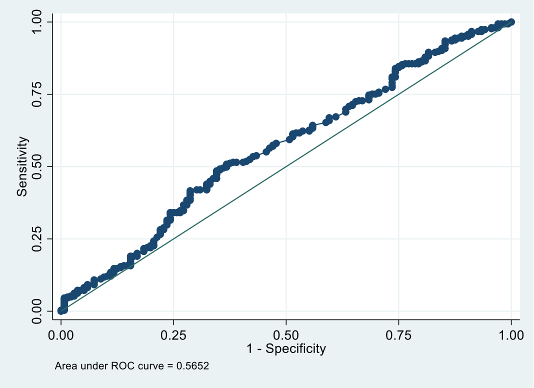

p = 0.014

OR 1.002 95%CI 1.0004 – 1.004; P = 0.02

Inspiratory total time:

OBS	Area	Std. Err	95% conf. interval
**410**	0.57	0.03	0.51 - 0.63

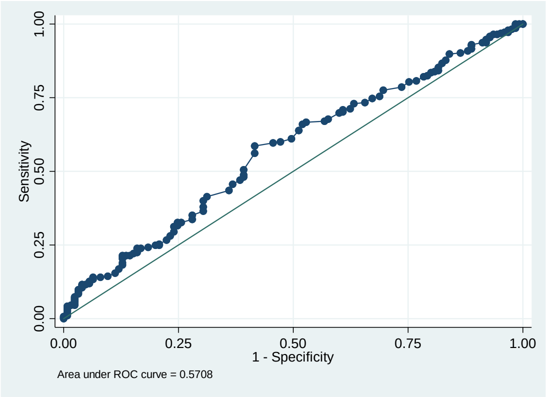

p = 0.010

OR 2.10 95%CI 1.14 – 3.87; P = 0.02

Rapid and shallow breathing index (RSBI):

OBS	Area	Std. Err	95% conf. Interval
**447**	0.48	0.03	0.42 - 0.54

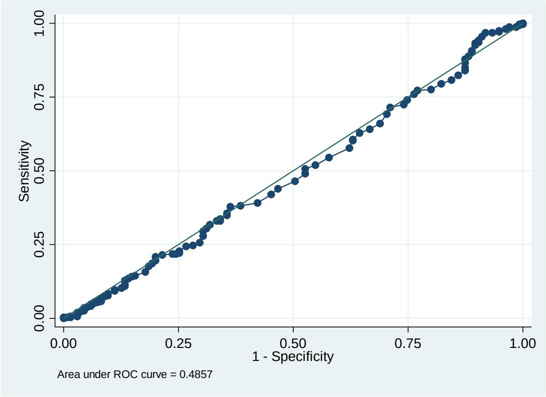

p = 0.317

The AUC-ROC was 0.48 with a confidence interval spanning the null value (0.5) and a non-significant difference from 0.5 (p = 0.317). This indicates that the RSBI lacks discriminative capacity to predict success in the extubation.

Diaphragmatic excursion

OBS	Area	Std. Err	95% conf. interval
**418**	0.53	0.03	0.46 - 0.59

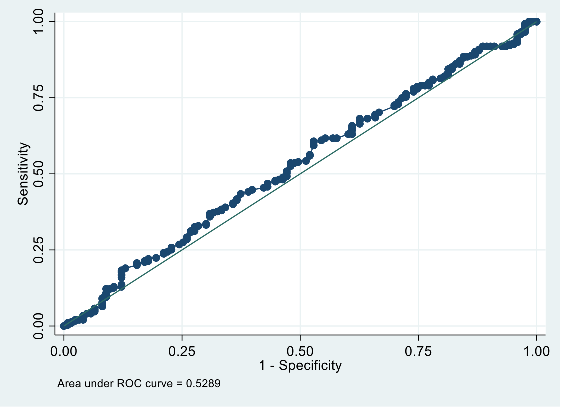

p = 0.176

The AUC-ROC was 0.53 with a confidence interval spanning the null value (0.5) and a non-significant difference from 0.5 (p = 0.176). This indicates that the diaphragmatic excursion lacks discriminative capacity to predict success in the extubation.

Duration of the diaphragmatic cycle

OBS	Area	Std. Err	95% conf. interval
**386**	0.53	0.03	0.47 - 0.60

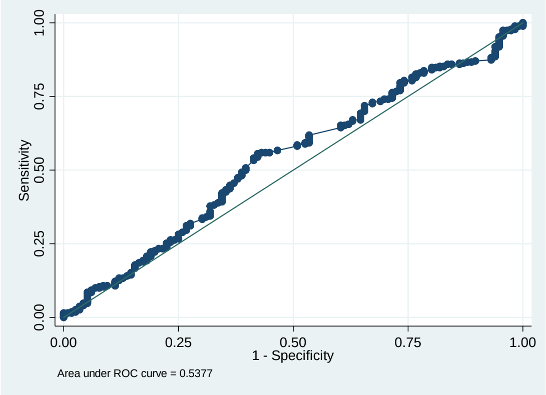

p = 0.120

The AUC-ROC was 0.53 with a confidence interval spanning the null value (0.5) and a non-significant difference from 0.5 (p = 0.120). This indicates that the duration of the diaphragmatic cycle lacks discriminative capacity to predict success in the extubation.

Diaphragmatic thickening index

OBS	Area	Std. Err	95% conf. interval
**353**	0.53	0.03	0.46 - 0.60

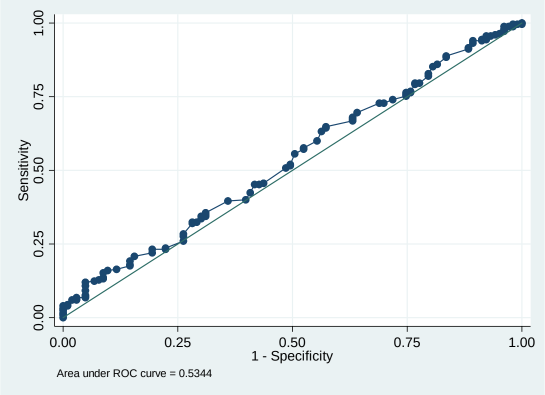

p = 0.384

The AUC-ROC was 0.53 with a confidence interval spanning the null value (0.5) and a non-significant difference from 0.5 (p = 0.384). This indicates that the diaphragmatic thickening index lacks discriminative capacity to predict success in the extubation.

Supplementary appendix. Interaction analysis.

Supplementary table 2a. Spontaneous breathing trial parsimonious model

SBT	Coef.	Std Err.	z	P>z	95% CI	
OMC	0.519	0.063	8.220	<0.001	0.395	0.643
VDC	-0.123	0.031	-3.960	<0.001	-0.184	-0.062
_cons	0.304	0.171	1.780	0.075	-0.031	0.639

Notes: SBT: Spontaneous breathing trial; OMC: Objective measurement of cough; VDC: Velocity of diaphragmatic contraction.
*R^2: 0.03

Supplementary table 2b. SBT model with the interaction variable

SBT	Coef.	Std. Err.	z	P>|z|	95% CI	
OMC	0.625	0.119	5.280	<0.001	0.393	0.857
VDC	-0.027	0.096	-0.280	0.782	-0.216	0.162
OMC x VDC	-0.040	0.038	-1.060	0.289	-0.115	0.034
_cons	0.054	0.291	0.190	0.852	-0.515	0.624

Notes: SBT: Spontaneous breathing trial; OMC: Objective measurement of cough; VDC: Velocity of diaphragmatic contraction.
*R^2: 0.03

The interaction variable (Cough x Velocity of diaphragmatic contraction) was not significantly associated with SBT and did not improve R^2 the of the model.

Supplementary table 2c. Evaluation of collinearity in the SBT predictor model with the interaction variable

Variable	VIF	SQRT VIF	Tolerance	R-Squared
OMC	3.58	1.89	0.279	0.720
VDC	12	3.46	0.083	0.916
OMC x VDC	15.13	3.89	0.066	0.933

Notes: VIF: variance inflation factor; SQRT VIF: The square root of variance inflation factor; OMC: Objective measurement of cough; VDC: Velocity of diaphragmatic contraction.

*The variance inflation factor was 15.13 and the tolerance 0.066, indicating collinearity induced by the interaction variable.

As the interaction variable (Cough x Velocity of diaphragmatic contraction) was not significantly associated with SBT, did not improve the R^2 of the model and induced collinearity, this variable was not introduced in the final model.

Supplementary table 2d. Successful extubation parsimonious model

Successful extubation	Coef.	Std. Err.	Z	P>z	95% CI	
SBT	5.646	0.254	22.18	<0.001	5.147	6.145
OMC	0.745	0.090	8.21	<0.001	0.567	0.923
VDC	-0.220	0.045	-4.87	<0.001	-0.309	-0.131
_cons	-4.692	0.346	-13.55	<0.001	-5.371	-4.014

Notes: SBT: Spontaneous breathing trial; OMC: Objective measurement of cough; VDC: Velocity of diaphragmatic contraction.
R^2: 0.55

Supplementary table 2e. Successful extubation predictor Model with the interaction variables

successful extubation	Coef.	Std. Err.	z	P>z	[95%	Conf.
SBT	4.718	0.528	8.930	<0.001	3.683	5.754
OMC	0.606	0.171	3.560	<0.001	0.272	0.941
VDC	-0.734	0.261	-2.810	0.005	-1.247	-0.222
OMC x VDC	0.051	0.054	0.970	0.334	-0.053	0.157
VDC x SBT	0.413	0.229	1.800	0.071	-0.036	0.862
_cons	-3.503	0.654	-5.350	<0.001	-4.786	-2.221

Notes: SBT: Spontaneous breathing trial; OMC: Objective measurement of cough; VDC: Velocity of diaphragmatic contraction.
R^2: 0.55
The interaction variables (OMC x VDC and VDC x SBT) were not significantly associated
with a successful extubation and did not improve R^2 the of the model

Supplementary table 2f. Evaluation of collinearity in the successful extubation predictor model with the interaction variables

Variable	VIF	SQRT VIF	Tolerance	R-Squared
SBT	4.350	2.090	0.230	0.770
OMC	3.700	1.920	0.271	0.730
VDC	13.580	3.680	0.074	0.926
OMC x VDC	15.520	3.940	0.065	0.936
VDC x SBT	7.040	2.650	0.142	0.858

Notes: VIF: variance inflation factor; SQRT VIF: The square root of variance inflation factor; SBT: Spontaneous breathing trial; OMC: Objective measurement of cough; VDC: Velocity of diaphragmatic contraction.

*The variance inflation factor was > 7 and the tolerance <0.15 for the interaction variables, indicating collinearity induced by the interaction variables.

As the interaction variables (OMC x VDC and VDC x SBT) were not significantly associated with SBT, did not improve the R^2 of the model, and induced collinearity, these variables were not introduced in the final model.

Supplementary appendix. Collinearity analysis.

Supplementary table 3a. Spontaneous breathing trial predictor model, collinearity analysis.

Variable	VIF	SQRT VIF	Tolerance	R-Squared
OMC	1.000	1.000	0.997	0.003
VDC	1.000	1.000	0.997	0.003

Notes: VIF: variance inflation factor; SQRT VIF: The square root of variance inflation factor; OMC: Objective measurement of cough; VDC: Velocity of diaphragmatic contraction.

*The variance inflation factor was 1 and the tolerance 0.99, indicating absence of collinearity

Supplementary table 3b. successful extubation predictor model, collinearity analysis.

Variable	VIF	SQRT VIF	Tolerance	R-Squared
SBT	1.030	1.020	0.968	0.032
OMC	1.030	1.020	0.970	0.030
VDC	1.010	1.000	0.991	0.009

Notes: VIF: variance inflation factor; SQRT VIF: The square root of variance inflation factor; SBT: Spontaneous breathing trial; OMC: Objective measurement of cough; VDC: Velocity of diaphragmatic contraction.

*The highest variance inflation factor was 1.03 and the lowest tolerance 0.96, indicating absence of collinearity
